# Exploring the protective effects of coenzyme Q10 on female fertility

**DOI:** 10.3389/fcell.2025.1633166

**Published:** 2025-08-29

**Authors:** Yu Jiang, Yao Han, Pengyun Qiao, Fa Ren

**Affiliations:** ^1^ Department of Reproductive Medicine, Affiliated Hospital of Shandong Second Medical University, Weifang, Shandong, China; ^2^ School of Clinical Medicine, Shandong Second Medical University, Weifang, Shandong, China

**Keywords:** CoQ10, female infertility, ROS, ART, oxidative stress

## Abstract

The global decline in fertility rates has intensified the reliance on assisted reproductive technology (ART) for infertility treatment. Antioxidant supplementation, particularly with coenzyme Q10 (CoQ10), has gained prominence as a therapeutic strategy to enhance fertility outcomes and ART success. As a lipid-soluble benzoquinone, CoQ10 plays dual roles in the metabolism of mitochondrial energy and antioxidant protection. By regulating oxidative stress and reducing reactive oxygen species (ROS), CoQ10 improves oocyte quality, ovarian function, and mitochondrial efficiency, thereby optimizing ART outcomes. Clinical studies demonstrate that CoQ10 supplementation enhances ovarian function, increases the number of eggs, and improves the quality of embryo, particularly in women with weak ovarian reserve functions or older age. This review synthesizes current evidence on CoQ10’s mechanisms in safeguarding female fertility, including its effects on oocyte maturation, embryonic development, and ovarian aging. Furthermore, it explores CoQ10’s potential in addressing conditions such as premature ovarian failure and polycystic ovary syndrome. This review provides an overview of CoQ10’s protective effect on female fertility and its potential clinical application in reproductive medicine, aiming to offer guidance for the future use of CoQ10 in ART clinical practice.

## Introduction

Based on the World Health Organization’s publication titled “Infertility Prevalence Estimation (1990-2021),” approximately 1/6 of the adult population worldwide, or 17.5%, suffers from infertility, with a rising trend. This growing prevalence has made infertility a significant public health concern (Herweck et al., 2025). Human reproduction depends on the production and transportation of gametes, fertilization, and development within the uterus, and any abnormalities in this process can negatively impact fertility and lead to infertility (Yong et al., 2021). Infertility is a medical condition that denotes a couple’s inability to become pregnant despite trying to conceive naturally for a duration of 1 year, without using any form of contraceptive measures (Tamrakar and Bastakoti, 2019). Available evidence suggests that approximately one in eight women between the ages of 15 and 49 seek infertility services (Carson and Kallen, 2021). Assisted reproductive technology (ART) is widely acknowledged as the most efficient solution for infertility. An increasing number of infertile couples are turning to ART to achieve pregnancy. ART encompasses all laboratory techniques involved in managing human oocytes, sperm, and embryos for the purpose of reproduction. This includes processes like ovarian stimulation, ovulation induction, *in vitro* fertilization (IVF), and the intracytoplasmic sperm injection (ICSI) method (Siristatidis et al., 2025). Despite significant advances in ART technology in recent years, the success rate of embryo implantation remains relatively low, making it difficult for infertile couples worldwide (Vidal et al., 2025). To address the low success rate of ART, it is essential to enhance the quality of gametes, which can subsequently improve clinical pregnancy rates. For instance, coenzyme Q10 (CoQ10), known for its antioxidant characteristics, shown promise in enhancing oocyte quality and boosting pregnancy rates (Alexandru et al., 2024), making it a prominent focus of current research.

CoQ10 is a crucial physiologically active substance present in various living organisms, exhibiting a range of biochemical properties, particularly its significant role in energy metabolism and antioxidant activity (Xu et al., 2025). The main biochemical characteristics of CoQ10 are as follows: 1. CoQ10 is an integral component of all cellular mitochondrial respiratory chains, participating in the processes of electron transfer and oxidative phosphorylation (López-Sánchez et al., 2025). It functions as an electron carrier within the inner mitochondrial membrane, facilitating the transfer of electrons from complexes (I, II) to complex III, thus promoting the synthesis of adenosine triphosphate (ATP). ATP is essential as the main energy source for cells, making CoQ10 vital for maintaining cellular energy metabolism (Rimle et al., 2025). 2. CoQ10 acts as a potent antioxidant, effectively scavenging oxygen free radicals within cells and protecting them from oxidative stress-related damage (Ahmadi et al., 2025). By neutralizing free radicals, it preserves the integrity of cellular structures and functions, thereby preventing oxidative damage to deoxyribonucleic acid (DNA), proteins, and lipids (Nesci et al., 2023). This antioxidant property is particularly critical in the field of reproductive medicine, as oxidative stress is a significant factor contributing to female infertility. 3. CoQ10 is a lipid-soluble benzoquinone characterized by a molecular structure that includes a quinone ring and a side chain made up of ten isoprene units. The structural configuration facilitates its integration into the cell membrane and the inner mitochondrial membrane, thereby enabling its electron transfer and antioxidant functions (López-Sánchez et al., 2025). 4. CoQ10 enhances immune response by facilitating the activity and functionality of immune cells (Roşian et al., 2025). This characteristic may improve the immune status of female infertility patients in reproductive medicine (Nie et al., 2023). Endogenous biosynthesis serves as the primary source of CoQ10. Key steps in this process include the formation of the benzoquinone ring and the assembly of the isoprenoid side chain. The rate-limiting enzyme, 4-hydroxybenzoate polyprenyltransferase, regulates the elongation of the side chain to ten isoprene units, which is characteristic of human CoQ10 (Shi et al., 2025). Importantly, while the human body can synthesize CoQ10, its synthesis capacity diminishes with age (Singh et al., 2007). Therefore, exogenous supplementation of CoQ10 holds significant clinical relevance.

Dietary intake serves as a secondary yet significant source of CoQ10, with rich sources including fatty fish, organ meats, nuts, and vegetable oils. While dietary contributions typically account for 10%–20% of total body CoQ10, this proportion increases in individuals with impaired biosynthesis, such as those with genetic mutations in COQ genes (Kuang et al., 2025). CoQ10 is primarily absorbed in the small intestine. Emerging evidence suggests that gut microbiota may play a significant role in the metabolism of CoQ10, although its precise function remains inadequately defined (Sun et al., 2025). Certain bacterial species, such as Eubacterium spp. and *Lactobacillus*, have been identified to synthesize CoQ homologs, which may be absorbed in the colon and subsequently converted into CoQ10 within host tissues (Liang et al., 2025). Once absorbed or synthesized, CoQ10 is distributed to tissues through lipoprotein transport, accumulating primarily in mitochondria, where it plays a crucial role in electron transport and ATP production (Deshwal et al., 2023). Understanding these sources and metabolic pathways provides a foundational rationale for CoQ10 supplementation in clinical contexts, particularly in states of deficiency—such as aging and ovarian dysfunction—where endogenous synthesis or dietary intake is insufficient to meet reproductive demands.

A study investigated the relationship between CoQ10 and female fertility and indicated that CoQ10 can support healthy oocyte development in women, prevent premature ovarian failure, and increase ovarian sensitivity to estrogen, ultimately leading to improved ovarian function (Brown and McCarthy, 2023). Importantly, studies have shown that CoQ10 can enhance the quality of eggs during *in vitro* fertilization and embryo transfer (IVF-ET) treatment, resulting in a higher success rate of embryo transfer (Zhang et al., 2020). Although CoQ10 has been used in clinical and human-assisted reproduction, its mechanism remains unclear and warrants further research and exploration. This review sought to collect and integrate existing knowledge of CoQ10 in reproductive medicine as a reference for future research into female fertility.

## CoQ10 in assisted reproductive technology

**FIGURE 1:**
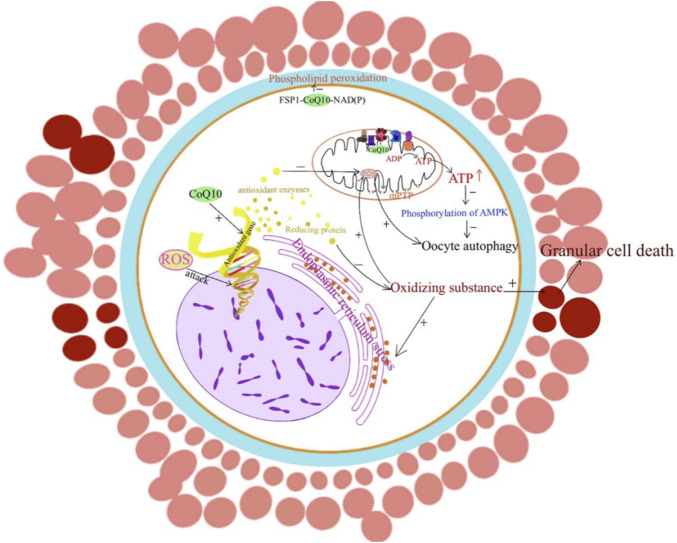
CoQ10 and improving oocytes quality. The mechanism by which CoQ10 affects oocytes is through the promotion of energy production, modulation of antioxidant gene expression levels, and involvement in various signaling pathways that inhibit oxidative stress and apoptosis.

CoQ10 supplementation has been linked to increased pregnancy rates in both natural conception and ART (Zhu et al., 2023). This phenomenon can be linked to its capacity to enhance gamete quality, which could help manage health issues often associated with IVF/ART (Zhu et al., 2023). A comprehensive review showed that oral CoQ10 supplementation (200 mg/thrice per day or 600 mg/day) increased clinical pregnancy rates in female infertility undergoing ART procedures (Florou et al., 2020). In addition, CoQ10 has been shown to enhance ovarian response and embryo quality to stimulation in young women with poor ovarian reserve during IVF-ICSI cycles (Xu et al., 2018). In this study, participants were given CoQ10 orally at a dosage of 200 mg three times a day for a period of 60 days (inclusion criteria: women age <35 years, antral follicle count <5, and anti-Mullerian hormone <1.2 ng/mL) (Xu et al., 2018). Studies vary in their use of CoQ10, but all results demonstrate the benefits of CoQ10 in the IVF-ICSI cycle. It is important to mention that although the elderly group receives a small dose of CoQ10, it is administered in conjunction with transcutaneous electrical acupoint stimulation (Qi et al., 2022). *In vitro* studies have demonstrated that the addition of 50 μM CoQ10 to the culture medium resulted in increased expression of Bcl2 and Sirt1 in cumulus cells, and a positive impact on the reduction of ROS, maturation rate, and first polar body extrusion was enhanced from 48.9% to 75.7% by the addition of CoQ10 to the culture medium (Lee et al., 2022). Additionally, CoQ10 treatment led to a significant reduction in the levels of apoptosis markers (Caspase3 and Bax) in both oocytes and cumulus cells and improved the distribution, relative mass, and membrane potential of mitochondria (Heydarnejad et al., 2019). CoQ10 primarily functions within mitochondria, but it can also be found in various other subcellular organelles, including the endoplasmic reticulum, Golgi, lysosome, and peroxisome. Its role in providing energy to oocytes and reducing oxidative stress is crucial (Figure 1). Oocytes contain a substantial number of mitochondria, making them the human cells with the highest mitochondrial density. Consequently, a significant amount of ATP is required for processes such as egg maturation, fertilization, and embryonic development (Nie et al., 2023). CoQ10 has been found to play a significant role in improving oocyte quality and increasing the clinical pregnancy rate of ART, which highlights the importance of CoQ10 in this process.

### CoQ10 and oocyte quality

Oocyte quality plays a crucial role in sperm-oocyte binding, early embryonic development, and improved clinical pregnancy rates. Unfortunately, various defects, such as oxidative stress and mitochondrial dysfunction, could hinder oocyte maturation (Li et al., 2023). Mitochondria serve as the energy factories of cells, and the development of eggs necessitates a substantial amount of ATP (Richani et al., 2021). CoQ10 is a crucial component of the mitochondrial electron transport chain, facilitating ATP production and enhancing mitochondrial function (Rimle et al., 2025). For women experiencing ovarian dysfunction or those of advanced age, CoQ10 supplementation may aid in restoring mitochondrial function and improving energy metabolism in oocytes (Zhang et al., 2019). The mechanisms of action of CoQ10 include restoring ROS imbalances and preventing DNA damage and oocyte apoptosis (Zhang et al., 2019). CoQ10 has been shown to enhance the maturation of inferior oocytes during meiosis, and 50 μM CoQ10 treatment could increase the average cell number of blastocysts (Yang et al., 2021). In addition, CoQ10 has been observed to significantly decrease the levels of oocyte apoptosis, double-strand breaks, and DNA damage (Hornos Carneiro et al., 2020). This decrease in aberrant cellular processes could result in lower levels of chromosome defects in diakinesis oocytes and aneuploidy in early embryonic divisions (Hornos Carneiro et al., 2020).

In addition, research has found that high levels of CoQ10 in the follicular fluid were associated with improved embryo morphokinetic parameters and higher pregnancy rates (Akarsu et al., 2017). In IVF, follicular fluid CoQ10 levels of A- and B-grade embryos were significantly higher than those of C- and D-grade embryos (Akarsu et al., 2017). The clinical trial results indicate that oral administration of 200 mg/day CoQ10 for 30–35 days could increase CoQ10 levels in follicular fluid, resulting in various benefits. This suggests that supplementing individuals in late reproductive stages with CoQ10 could improve the quality of mature oocytes and increase the rate of successful fertilization (Giannubilo et al., 2018). Research has shown that supplementation with CoQ10 can improve the *in vitro* maturation rate of immature oocytes in patients with endometriosis compared to those who do not receive the supplement (Romero et al., 2020). Supplementation with 50 μM CoQ10 during *in vitro* maturation has been found to increase oocyte maturation rates and decrease postmeiotic aneuploidies in women aged 38-46 (Ma et al., 2020). The specific mechanism warrants further exploration. CoQ10 enhances oocyte maturation by stabilizing mitochondrial membrane potential, augmenting ATP production through increased electron transfer from complexes I and II to III in the respiratory chain (Bozdemir et al., 2025). Additionally, it upregulates anti-apoptotic proteins such as Bcl2 while downregulating pro-apoptotic factors like Caspase3 and Bax. Notably, the relative expression level of GDF9, an oocyte-specific growth factor, is significantly increased (Heydarnejad et al., 2019). In addition, CoQ10 reduces the mRNA expression of HSD11B1 and FKBP5 in immature oocytes, as well as in cumulus cells regarding HSD11B1. Additionally, it affects the Bax:Bcl2 mRNA expression ratio (Ruiz-Conca et al., 2022). The potential mechanism by which CoQ10 improves the quality deterioration of aging-related oocytes is closely associated with its antioxidant properties, enhancement of mitochondrial function, promotion of autophagy, and inhibition of apoptosis. These biological processes are regulated by signaling pathways such as PPAR, TNF, and MAPK (Yang et al., 2022). *In vitro* studies have shown that a concentration of 50 μM CoQ10 in culture media elevates the expression of Sirt1, a key regulator of mitochondrial function, in cumulus cells. This elevation reduces ROS-induced DNA damage and improves the rates of first polar body extrusion from 48.9% to 75.7% (Lee et al., 2022). During oocyte aging, the levels of proteins associated with mitochondrial biosynthesis, specifically SIRT1 and PGC-1α, as well as those involved in mitochondrial phagocytosis, namely, PINK1 and PARKIN, are diminished. However, the *in vitro* supplementation of CoQ10 has been shown to mitigate the decline of these proteins attributable to aging (Niu Y. et al., 2020). These findings suggest that using CoQ10 for oocyte culture could be a beneficial option for improving oocyte quality, providing clinicians with more treatment options to explore.

### CoQ10 and ovarian aging

The process of ovarian aging is marked by a gradual decline in mitochondrial function, which affects both somatic and germ cells in the ovary (Colella et al., 2021). This decline can lead to lower fertilization rates and hinder embryonic development (Labarta et al., 2019). The negative impact of aging on ovarian reserve, chromosome segregation, and embryonic capacity is attributed to decreased mitochondrial function and energy production (Meldrum et al., 2016). The decline in CoQ10 levels is prevalent among individuals aged 35 and older, and it appears to coincide with the age-related decrease in fertility and an increase in the rate of embryonic aneuploidy. This suggests that the reduction in CoQ10 expression is linked to ovarian aging (Miles et al., 2004). Interestingly, a study on aging mice demonstrated that CoQ10, a mitochondrial cofactor, can effectively reverse most of these changes (Meldrum et al., 2016). A study has shown that the use of CoQ10 during the oocyte growth phase has a significant rejuvenating effect on aging oocytes and can counteract ovarian aging (Homer, 2021). Additionally, CoQ10-treated mice showed improved ovarian function. This was attributed to an increase in the activity of superoxide dismutase, glutathione peroxidase, and glutathione S-transferase, while also reducing malondialdehyde and ROS content (Honardoust et al., 2021). These findings suggest that CoQ10 may have a protective effect against ovarian ischemia‒reperfusion damage. In a study investigating the molecular mechanisms of ovarian aging, the data available suggest that oxidative stress is the primary cause. Nevertheless, the use of the direct antioxidant CoQ10 has been found to alleviate ovarian aging (Tesarik et al., 2021). In the context of ovarian aging, CoQ10 mitigates mitochondrial dysfunction by activating superoxide dismutase and glutathione peroxidase, which reduces malondialdehyde levels and the accumulation of ROS (Honardoust et al., 2021; Tekin et al., 2024). During aging, there is a decrease in mitochondrial biogenesis-related proteins (SIRT1 and PGC-1α) and mitophagy-related proteins (PINK1 and PARKIN). The addition of CoQ10 effectively prevents the age-related decline of these proteins (Niu Y. et al., 2020). This intervention not only preserves the primordial follicle reserve but also enhances ovarian sensitivity to gonadotropins, as evidenced by improved ovarian responses in women with diminished ovarian reserve following CoQ10 pretreatment (Teppa-Garrán et al., 2025). The recommended dosage of CoQ10 varies among individuals. Research indicates that supplementing with CoQ10 at a dosage of 600 mg per day for 60 days can enhance ovarian response in women with diminished ovarian reserve. During *in vitro* fertilization, this supplementation may reduce the demand for gonadotropins, thereby improving the response to follicle pre-stimulation (Gat et al., 2016). As a result, restoring CoQ10 levels could prove to be an effective intervention for ovarian anti-aging.

### CoQ10 and embryonic development

CoQ10 not only positively impacts egg quality and ovarian function but also enhances the developmental potential of embryos. It improves embryo quality by reducing oxidative stress and enhancing mitochondrial function, which in turn increases embryo implantation and pregnancy rates (Shang et al., 2024). Embryo development commences with the formation of the zygote, progressing through the morula and blastula stages (Coticchio et al., 2019). With advancements in culture conditions, embryos can now survive *in vitro* for extended periods. The physiological level of ROS is crucial for embryonic development. However, excessive ROS production can adversely affect the embryo by inducing intracellular damage and metabolic disorders (Niu Y. J. et al., 2020). Mitochondria play a vital role in several cellular processes, such as ATP production, cytoplasmic redox regulation, calcium homeostasis, signal transduction, and apoptosis. The proper functioning of mitochondria is considered essential for the early stages of embryonic development (Marei et al., 2019). Research has indicated that CoQ10 has the potential to safeguard the structure of mitochondria and facilitate the development of the nucleus of oocytes, leading to a higher average cell count of blastocysts (Yang et al., 2021). Animal studies have demonstrated that the supplementation of CoQ10 *in vitro* during the culture of embryos significantly enhances the cleavage rate, blastocyst formation rate, hatching rate, and blastocyst expansion rate of early embryos (Ben-Meir et al., 2015; Stojkovic et al., 1999). A prospective randomized controlled trial has shown that pretreatment with CoQ10 can significantly increase the number of high-quality early embryos in young women who have a decreased ovarian reserve and a low prognosis (Xu et al., 2018). Women in the CoQ10 group demonstrated an increased number of retrieved oocytes, a higher fertilization rate, and a greater quantity of high-quality embryos. Moreover, significantly fewer women treated with CoQ10 experienced cancelled embryo transfers due to poor embryo development compared to the control group (8.33% vs 22.89%). Additionally, a higher percentage of women in the treatment group had available cryopreserved embryos (18.42% vs 4.3%) (Xu et al., 2018). In addition, CoQ10 led to a decrease in the rate of embryonic apoptosis and an improvement in quality parameters associated with embryo development and hatchability (Higa et al., 2018; Kalantar et al., 2019; Shin et al., 2024). CoQ10 has been identified as a potential enhancer of embryonic development, having a positive impact on early embryonic maturity, energy supply, and divisive ability. These findings suggest a promising new approach for improving the quality of embryonic development.

CoQ10, an important antioxidant and a key component of cellular energy metabolism, has garnered significant attention in the field of reproductive health in recent years. Its role in enhancing egg and embryo quality, as well as increasing pregnancy rates, is particularly noteworthy. Table 1 summarizes the studies evaluating CoQ10 in relation to female infertility, as discussed in this study. In clinical practice, the dosage and duration of CoQ10 supplementation for female patients undergoing ART or experiencing fertility concerns should be individualized, taking into account factors such as ovarian reserve, age, and specific reproductive conditions. For women with normal ovarian reserve undergoing standard IVF/ICSI cycles, a daily oral dose of 200 mg of CoQ10, administered for 30–35 days prior to ovarian stimulation, is advised. This regimen is intended to elevate follicular fluid CoQ10 levels, improve oocyte maturation rates, and enhance early embryo quality (Giannubilo et al., 2018). For patients with diminished ovarian reserve, a higher dosage of 600 mg/day over a period of 60 days has been shown to be more effective in enhancing ovarian response to gonadotropins. This approach not only reduces the required dosage of gonadotropins but also increases the number of retrieved oocytes and high-quality embryos (Xu et al., 2018; Florou et al., 2020). Women aged 35 years and older, as well as those experiencing premature ovarian failure, may benefit from long-term supplementation of CoQ10. Specifically, a dosage of 200 mg/day for a duration of 90 days, combined with transdermal acupoint electronic therapy, is recommended. This supplementation aims to counteract the age-related decline in endogenous CoQ10 synthesis, restore mitochondrial function in oocytes, and reduce postmeiotic aneuploidies (Qi et al., 2022). *In vitro* studies support the adjunctive use of 50 μM CoQ10 in maturation media during *in vitro* maturation (IVM) cycles, which may further enhance maturation rates and mitigate oxidative stress-induced DNA damage (Ma et al., 2020).

**TABLE 1:** Summary of studies evaluating CoQ10 effects on female fertility**.** POR: poor ovarian response; FF: follicular flfluid; IVM: *in vitro* maturation; POI: premature ovarian insufficiency.

Research object	PatientsNumber	Age (years) mean ± SD	CoQ10 Treatment	Main Results	Year	References
Infertile women	n = 15	31-41	200 mg/day (30 days)	Increases in follicular content	2018	Giannubilo et al.
POR women	n = 185	32.27 ± 3.47	10 mg/three times a day (a total duration of 3 months)	Increase the numbers of retrieved eggs Improves clinical pregnancy rate	2019	Zhang et al.
Infertile women	n = 45 (age ≥ 38) n = 45 (age ≤ 30)	39.8 ± 2.0 (age ≥ 38) 26.8 ± 2.1 (age ≤ 30)	*In vitro* addition (IVM media for 24–48 h, 50 mmol/L)	Increases oocyte maturation rates (≥38) Reduces postmeiotic aneuploidies (≥38)	2020	Ma et al.
POR women	n = 110	39.22 ± 2.94	10 mg/three times a day (a total duration of 3 months)	Increase the numbers of retrieved eggs Increase superior embryos Improves endometrial receptivity	2022	Qi et al.
POI women	n = 32	<40	——	Inversely associated with POI A protective factor for ovarian	2023	Ma et al.
Infertile women	n = 34	35.38 ± 3.46	500 IU twice daily for 12 weeks	High good-quality embryo rate High implantation rate High clinical pregnancy rate	2024	Shin et al.

Clinical data indicate that the safe daily dosage of CoQ10 for humans is 1,200 mg/person, which significantly exceeds the conventional dosage (Arenas-Jal et al., 2020). Research has demonstrated that CoQ10 exhibits low toxicity, lacks potential genetic toxicity, and does not induce serious adverse reactions in humans (Arenas-Jal et al., 2020). Furthermore, CoQ10 does not interfere with endogenous synthesis nor does it accumulate in plasma. However, gastrointestinal symptoms may arise when the daily dosage surpasses 1,200 mg (Villalba et al., 2010). It is important to note that the safety of this compound in children, pregnant women, and lactating women has not been thoroughly validated.

### CoQ10 and female fertility

**FIGURE 2:**
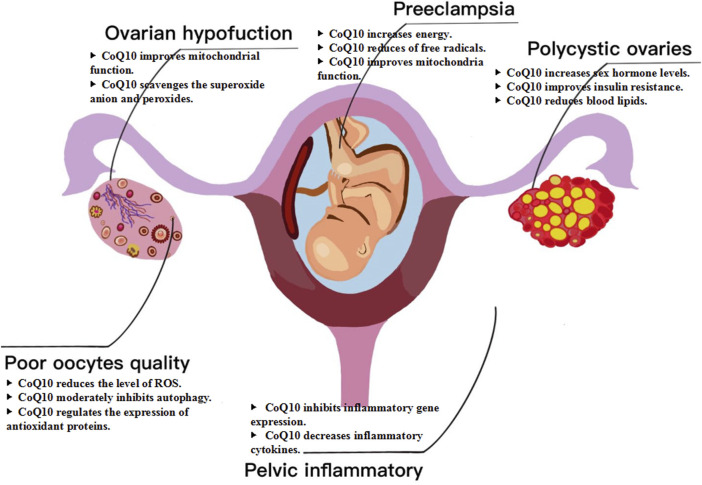
Mechanism of CoQ10 for female fertility. CoQ10 has emerged as a potential intervention for female infertility, specifically in cases related to ovarian dysfunction, poor oocyte quality, polycystic ovaries, pelvic inflammatory disease, and other factors.

CoQ10 has emerged as a potential intervention for female infertility, specifically in cases related to ovarian dysfunction, poor oocyte quality, polycystic ovaries, pelvic inflammatory disease, and other factors (Nie et al., 2023; Sarrible et al., 2025). CoQ10 is a potent antioxidant that neutralizes free radicals and reduces oxidative stress damage to ovarian tissue. This is particularly significant for patients with premature ovarian failure, as oxidative stress is a critical factor contributing to ovarian dysfunction. Supplementation with CoQ10 can effectively protect ovarian function and delay the aging process of the ovaries (Ma et al., 2023). Polycystic ovary syndrome (PCOS) is a prevalent endocrine disorder that can cause subfertility and obstetric complications (Zhang et al., 2025). Studies have shown that CoQ10 can impede the progression of the disease and enhance fertility in women with PCOS (Zhang et al., 2023). Specifically, studies have demonstrated that CoQ10 can raise follicle-stimulating hormone levels and lower testosterone levels, as well as decrease HOMA-IR, FINS, and FPG. CoQ10 has been observed to decrease triglycerides, total cholesterol and low-density lipoprotein cholesterol while increasing high-density lipoprotein cholesterol in women with PCOS (Izadi et al., 2019). For patients with diminished ovarian reserve, the supplementation of CoQ10 is particularly crucial, as it aids in restoring the energy levels of oocytes and enhancing their quality. Research indicates that CoQ10 can improve mitochondrial function in oocytes, reduce ROS levels, and regulate antioxidant proteins and autophagy, thereby augmenting the fertilization capacity and developmental potential of embryos (Feng et al., 2022). Inflammatory diseases represent a significant category of disorders that impair the function of the female reproductive system, with pelvic inflammatory disease serving as the most prominent example (Dong et al., 2025). CoQ10 has been shown to effectively reduce inflammatory cytokines by inhibiting their gene expression (Mantle et al., 2021). A meta-analysis of seventeen randomized controlled trials demonstrated that CoQ10 can significantly reduce the levels of CRP, IL-6, and TNF-α (Fan et al., 2017). Although there are no clinical trials or related studies on the use of CoQ10 in treating female pelvic inflammatory diseases, it has been proven to reduce the inflammatory response (Abiri and Vafa, 2021). Additionally, supplementation with CoQ10 has been found to be an effective intervention in reducing the risk of developing preeclampsia. This condition is associated with abnormal placental development due to several factors. Interestingly, the reduction of free radicals through CoQ10 can help mitigate this risk (Garrido-Maraver et al., 2014; Teran et al., 2018). In summary, CoQ10 supplementation has demonstrated considerable potential in enhancing female fertility, particularly by improving ovarian function, increasing egg quality, and mitigating inflammatory responses (Figure 2).

Despite numerous positive reports regarding the role of CoQ10 in enhancing female fertility, it is equally important to conduct a critical analysis. For example, a total of 169 women of childbearing age were evaluated, comprising 76 who received CoQ10 treatment and 93 in the control group. Compared to the control group, there were no significant differences in the clinical pregnancy rate or live birth rate among women receiving CoQ10 treatment for each embryo transfer or complete stimulation cycle (Xu et al., 2018). Similarly, the study found no significant difference in clinical pregnancy rates among 78 IVF cycles following oral administration of CoQ10 (Gat et al., 2016). This finding may be attributed to the limitations associated with small sample sizes in clinical research. Numerous studies have demonstrated the benefits of CoQ10 for female fertility; however, many of these studies have small sample sizes, typically involving fewer than 50 participants (Nie et al., 2023). Such limited sample sizes diminish the statistical power of the studies, increasing the likelihood of overlooking actual effects or drawing inaccurate conclusions.

### CoQ10 in combination therapy

The application of CoQ10 in reproductive medicine is significantly enhanced when used in conjunction with adjunct therapies. This synergistic effect amplifies its therapeutic potential. When combined with vitamin E, a lipid-soluble antioxidant, CoQ10 provides improved protection for oocytes against oxidative damage. Research indicates that vitamin E safeguards cell membranes from lipid peroxidation, while CoQ10 mitigates mitochondrial ROS. Together, these compounds improve the maturation rate of oocytes and the cleavage rate of embryos (Maddahi et al., 2024). In clinical studies, CoQ10, when combined with vitamin E, has been shown to improve levels of fasting blood sugar, insulin, homeostasis model assessment of insulin resistance, sex hormone-binding globulin, and total testosterone in patients diagnosed with PCOS (Izadi et al., 2019). Animal studies indicated that, compared to the vitamin C group, the group supplemented with vitamin E and CoQ10 exhibited significantly higher maturation and cleavage rates (Maddahi et al., 2024). Furthermore, the combination of CoQ10 and melatonin has been shown to increase the number of oocytes collected (Shang et al., 2024). In an *in vitro* study involving cattle, the combined administration of CoQ10, IGF1, and melatonin significantly enhanced oocyte quality and functionality. This improvement was achieved by ROS levels and apoptosis, increasing mitochondrial membrane potential, optimizing mitochondrial distribution, and upregulating genes associated with oocyte quality (Zhang et al., 2025). Interestingly, clinical studies have demonstrated that the combined application of dehydroepiandrosterone (DHEA) and CoQ10 in patients with diminished ovarian reserve can significantly enhance the number of antral follicles and improve ovarian responsiveness during intrauterine insemination and IVF cycles. However, this combination appears to have no effect on clinical outcomes (Gat et al., 2016). In addition to its use alongside antioxidants, CoQ10 combined with transcutaneous electrical acupoint stimulation pretreatment significantly enhances ovarian responsiveness, increases the number of retrieved eggs and high-quality embryos, and improves endometrial receptivity in patients with ovarian hyporesponsiveness (POR). However, the administration of CoQ10 alone does not yield such significant effects (Qi et al., 2022). Hence, the combined application of CoQ10 alongside other adjunctive treatments, such as vitamins, antioxidants, and acupuncture, significantly enhances female egg quality and ovarian function. Future research should focus on further investigating the synergistic effects of CoQ10 in conjunction with other therapeutic modalities.

### Future research directions and unresolved questions

Despite the increasing body of evidence supporting the role of CoQ10 in enhancing female fertility, several critical gaps and future research directions must be emphasized to facilitate its clinical translation. First, mechanistic investigations require a higher degree of precision that transcends generalized antioxidant and mitochondrial effects. Current studies have demonstrated that CoQ10 can enhance oocytes quality by exerting antioxidant effects and improving mitochondrial function (Chen et al., 2025). However, the specific mechanisms underlying its action remain inadequately understood. For instance, how does CoQ10 precisely regulate the energy metabolism of oocytes? Is its antioxidant effect mediated through specific signaling pathways? These questions warrant further investigation through molecular biology and cell biology experiments. Secondly, the dosage and treatment duration of CoQ10 vary significantly across different studies, and there is currently no standardized protocol. Future research should aim to establish the optimal dosage and treatment duration tailored to specific populations, such as age and ovarian reserve function status, in order to maximize therapeutic efficacy while minimizing potential side effects. Thirdly, the combined application of CoQ10 and other nutrients, such as melatonin (Zhang and Wu, 2025), may yield synergistic effects. However, numerous unresolved questions and avenues for exploration remain in this field. Future research should investigate the absorption variations of different CoQ10 dosage forms when combined with other nutrients, while also considering individual differences, including age and ovarian reserve. Furthermore, it is recommended to prioritize research on specific combination strategies tailored to various reproductive disorder phenotypes, such as premature ovarian failure and oocyte maturation disorders.

## Conclusion

CoQ10, recognized as a natural antioxidant and regulator of energy metabolism, plays a crucial role in enhancing the quality of oocytes and embryos, increasing pregnancy rates, and safeguarding ovarian function. It demonstrates significant potential in the treatment of infertility; however, numerous research gaps remain that require urgent attention. A comprehensive exploration of its mechanisms of action, optimization of dosage and treatment protocols, evaluation of combined application effects, and expansion of research scope are essential to further elucidate the role of CoQ10 in infertility treatment, thereby providing effective therapeutic strategies for a broader patient population. Future research should emphasize interdisciplinary collaboration, integrating basic research with clinical practice to advance the application and development of CoQ10 in reproductive health.

## Abbreviations

ART, assisted reproductive technology; CoQ10, coenzyme Q10; ROS, reactive oxygen species; IVF, *in vitro* fertilization; ICSI, intracytoplasmic sperm injection; MAPK, mitogen-activated protein kinase; PCOS, polycystic ovary syndrome; RCT, randomized controlled trial; POR, poor ovarian response; FF, follicular flfluid; HCG, human chorionic gonadotrophin; IVM, *in vitro* maturation; POI, premature ovarian insufficiency; HOMA-IR, homeostatic model assessment of insulin resistance.
